# Druggability of cavity pockets within SARS-CoV-2 spike glycoprotein and pharmacophore-based drug discovery

**DOI:** 10.2217/fvl-2020-0394

**Published:** 2021-06-01

**Authors:** Alireza Mohebbi, Fatemeh Sana Askari, Ali Salehnia Sammak, Mohsen Ebrahimi, Zahra Najafimemar

**Affiliations:** 1 ^1^Department of Microbiology, School of Medicine, Golestan University of Medical Sciences, Gorgan 4934174515, Iran; 2 ^2^Student Research Committee, School of Medicine, Golestan University of Medical Sciences, Gorgan 4934174515, Iran; 3 ^3^Department of Microbiology, Faculty of Basic Sciences, Rasht Branch, Islamic Azad University, Rasht, Gilan 4147654919, Iran; 4 ^4^Children's Research Center, Golestan University of Medical Sciences, Gorgan, Iran

**Keywords:** COVID-19, docking, SARS-CoV-2, spike protein, virtual screening

## Abstract

**Aim:** Virus spike glycoprotein of SARS-CoV-2 is a good target for drug discovery. **Objective:** To examine the potential for druggability of spike protein for pharmacophore-based drug discovery and to investigate the binding affinity of natural products with SARS-CoV-2 spike protein. **Methods:** Druggable cavities were searched though CavityPlus. A pharmacophore was built and used for hit identification. Autodock Vina was used to evaluate the hits' affinities. 10 chemical derivatives were also made from the chemical backbone to optimize the lead compound. **Results:** 10 druggable cavities were found within the glycoprotein spike. Only one cavity with the highest score at the binding site was selected for pharmacophore extraction. Hit identification resulted in the identification of 410 hits. **Discussion:** This study provides a druggable region within viral glycoprotein and a candidate compound to block viral entry.

## Graphical abstract

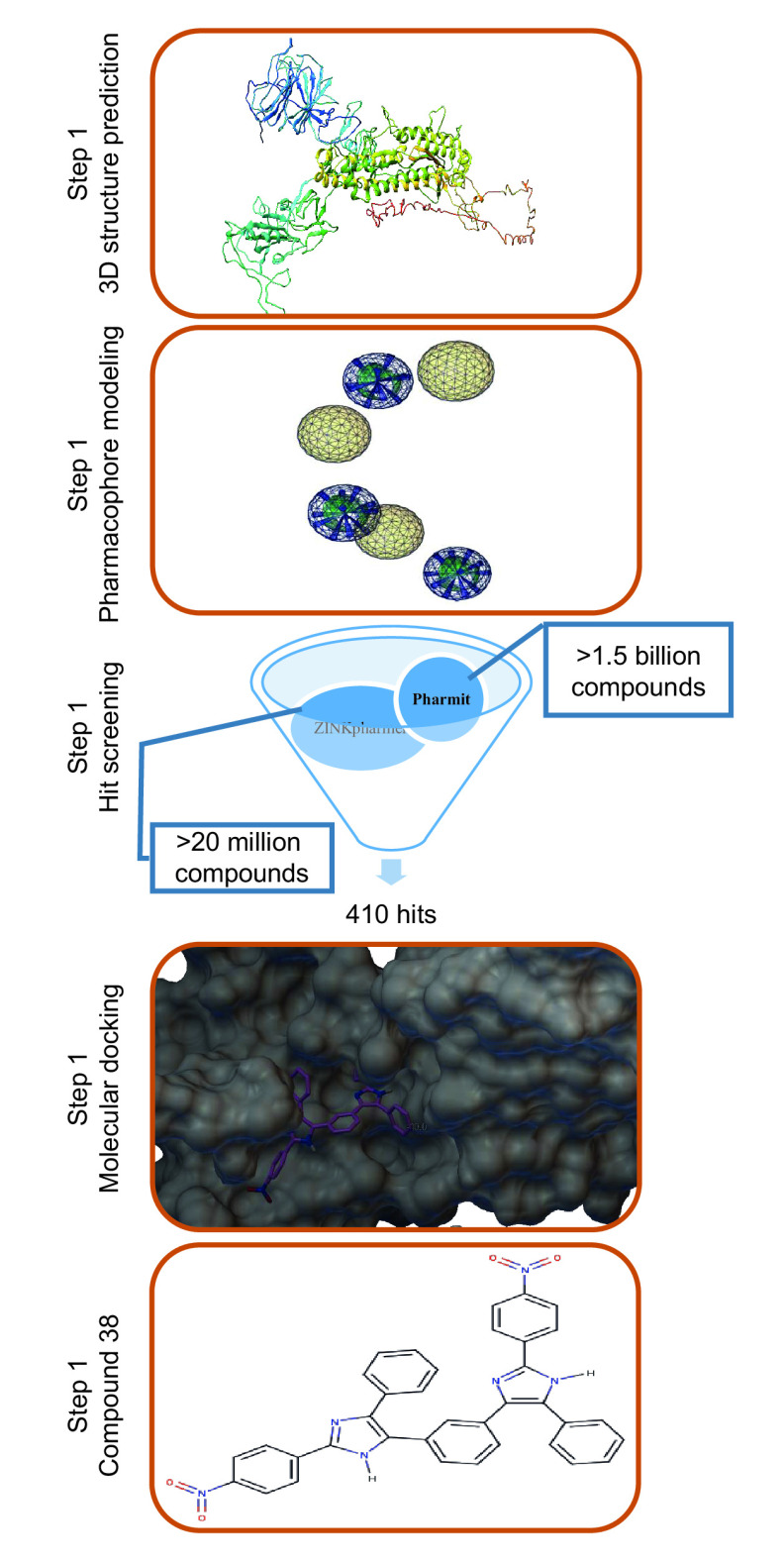

In December 2019, a viral disease called coronavirus disease-2019 or COVID-19 broke out in China, causing the deaths of hundreds of thousands of people. In several countries, the virus has spread rapidly and death rates are still rising [1–3]. The etiological agent of the pandemic is named severe acute respiratory syndrome coronavirus 2 (SARS-CoV-2). SARS-CoV-2 is a positive single-stranded RNA virus comprising two classes of structural proteins and nonstructural proteins (NSPs) [4,5]. Infection begins by binding the structural spike (S) protein to the host cell surface ACE2 receptor [6]. S glycoprotein is important for binding and entry and plays a determining role in SARS-CoV-2 host tropism and pathogenesis [7–10].

The diagnosis of COVID-19 is required to determine the exact number of patients that must be treated. Viral gene detection by real-time polymerase chain reaction (RT-PCR) is currently the most reliable SARS-CoV-2 detection method [11]. Further diagnostic methods of COVID-19, including antibody/antigen and other sensitive approaches are reviewed by Yüce *et al.* [12]. Researchers identified several viral proteins in SARS-CoV-2 as therapeutic targets, including S protein, envelope (E) protein, membrane (M) protein, nucleocapsid protein (N), proteases (3CLpro and PLpro), Nsp1, Nsp3 (Nsp3b, Nsp3c, Nsp3e), Nsp7 Nsp8 complex, Nsp9–Nsp10 and Nsp14–Nsp16, ORF7a, helicase and RdRp [13,14]. Among them, the S protein gained more attention due to its role in the entry of the virus. S protein contains two domains S1 and S2 [15]. The S1 domain has a significant role to play in virus entry. Viral entrance continues with interactions between human receptor ACE2 and S1 receptor-binding domain (RBD) of S protein [5,16–19]. In addition, the S2 domain induces the fusion of viral membranes with the host. This protein may therefore be considered as an essential target for developing new treatments [15,20–22].

As noted, comprehensive studies have been performed by researchers to identify effective therapeutic targets of SARS-CoV-2. Given the advantages of identifying promising targets, some progress has been done *in vitro*. In this regard, active nucleos(t)ide analog triphosphate remdesivir (GS-443902) has been shown to interfere with SARS-CoV-2 nonstructural protein 12 (nsp12) polymerase and causes the termination of RNA synthesis [23]. Previously, Xia *et al.* utilized HCoV-derived peptide OC43-HR2P to show heptad repeat (HR) 1 region of S glycoprotein is a suitable target site for inhibition of HCoVs [24]. Recently, this team has shown an optimized peptide named EK1C4 by forming a stable conformation has membrane fusion inhibitory activity in multiple *Coronavirus* species, especially SARS-CoV-2 [25]. Additionally, results of recent trials showed that combination of remdesivir with baricitinib reduces time to recovery in hospitalized patients with COVID-19 [26]. Furthermore, a broad-spectrum promising anti-viral agent is the anti-malarial and autoimmune disease drug named chloroquine, interferes with the SARS-CoV-2 infection at both entry, and at post-entry stages [27]. Concerning the neutralizing immunotherapy for preventing viral infections, Phase III trials of baricitinib are in progress in patients with COVID-19 infection by Eli Lilly (USA) [26]. Additionally, Regeneron Pharmaceuticals is continuing with Phase III trials on patients for assessing efficacy and safety of sarilumab (SAR153191) for hospitalized patients with COVID-19 (ClinicalTrials ID: NCT04327388). Furthermore, bamlanivimab, casirivimab (REGN10933) and imdevimab (REGN10987) were authorized for emergency use by the US FDA [28].

The use of *in silico* approaches may be a good option in the current situation. This can reduce the time and cost of drug discovery. In addition, *in silico* tools will contribute to the simplification of hit identification, impact on lead development, improvement in absorption, distribution, metabolism, excretion and toxicity and therefore lead to the identification of an efficient and safe-to-use drug-like compounds [29]. Using computational methods, researchers evaluated the various compounds in order to determine the effectiveness of these compounds in the treatment of COVID-19 disease [30–35]. However, the findings only supported the concept of FDA-approved anti-viral agents with the same targets could have similar results for COVID-19.

Considering S glycoprotein as the main structural protein with key roles at the early viral life cycle, targeting S glycoprotein can significantly reduce viral infection and subsequent disease symptoms by blocking entry and subsequent replication of SARS-CoV-2. The goal of this study was to computationally analyze SARS-CoV-2 S glycoprotein for its potential druggable cavities and to establish an efficient pharmacophore for anti-viral drug discovery. In addition, a massive virtual library of natural products was screened to suggest chemical candidates to block SARS-CoV-2 entry.

## Materials & methods

### Preparation of SARS-CoV-2 spike glycoprotein

Prediction of 3D protein structures from amino acid sequence SARS-CoV-2 glycoprotein (accession number: YP_009724390.1) was performed by using the I-TASSER server (http://zhang.bioinformatics.ku.edu/I-TASSER) [36]. In comparison with homology-based tools, I-TASSER server generates full-length 3D protein structure that enables one to make amino acid changes. The best model with highest confidence score was used for the study. The cavity with highest drugability score was used for further studies.

### Finding druggable cavity pockets of SARS-CoV-2 spike glycoprotein

Predicted 3D structure of SARS-CoV-2 Spike glycoprotein used for analysis of its binding cavities by using CavityPlus (www.pkumdl.cn/cavityplus) server [37]. CAVITY module was used to detect potential binding sites on the surface of the given protein structure. The druggable cavity with the highest drug score was used for pharmacophore modeling with CavPharmer module.

### Optimization of pharmacophore features & hit identification

The resulting pharmacophore feature.mol2 file was manually modified to increase chance of hit identification. Briefly, excluded volume center features were removed from the file. Additionally, positive/negative electrostatic centers were reduced to one. Further features including h-bond donor/acceptor and hydrophobic centers both were reduced to minimum three. Two screening servers ZINCPharmer [38] (206,433,075 conformers of 21,777,093 compounds) and Pharmit [39] (1,599,077,712 conformers of more than 300 million compounds) were used for pharmacophore-matched hit identification. Briefly, pharmacophore features file was uploaded in each server and it was screened for shape-matched hits. Hits were checked for duplication by using OpenBabel [40] software command line.

### Molecular docking & hit optimization

Affinity of identified hits was further investigated by using molecular docking. For this purpose, the open-source tool Autodock Vina [41] was used in the setting of PaDEL-ADV (www.yapcwsoft.com/dd/padeladv/). Vina is a fast and accurate tool for ligand-receptor docking and with PaDEL-ADV it allows high-throughput screenings of several ligands in one run. Because of large structure of SARS-CoV-2 Spike glycoprotein, docking was performed on the predicted druggable sites only. In this regard, the monomeric viral glycoprotein was treated as receptor in the MGLTools 1.5.6 software (Molecular Graphics Laboratory, The Scripps Research Institute). Accordingly, a grid-box was defined in 3D dimension to encompass the entire cavity. The gridbox center coordinates were 119.371, 145.957, and 141.39 along x, y, and z axis, respectively. The number of grid points and the spacing were kept to default values.

### Lead optimization

Compound(s) with higher affinity to SARS-CoV-2 spike glycoprotein were further optimized by ChemT software [42]. For this purpose, a functional group (R^1^) was chosen based on interaction analysis of the compound(s) and receptor. R^1^ group was substituted with the software pre-existing ten functional groups for building template-based chemical libraries (Supplementary material, Library.sdf). Autodock Vina was used to further screen the library for evaluation of their affinities to the receptor. Chemicals descriptor's values were kept as default.

## Results

### S protein druggable cavities

**Figure 1.**
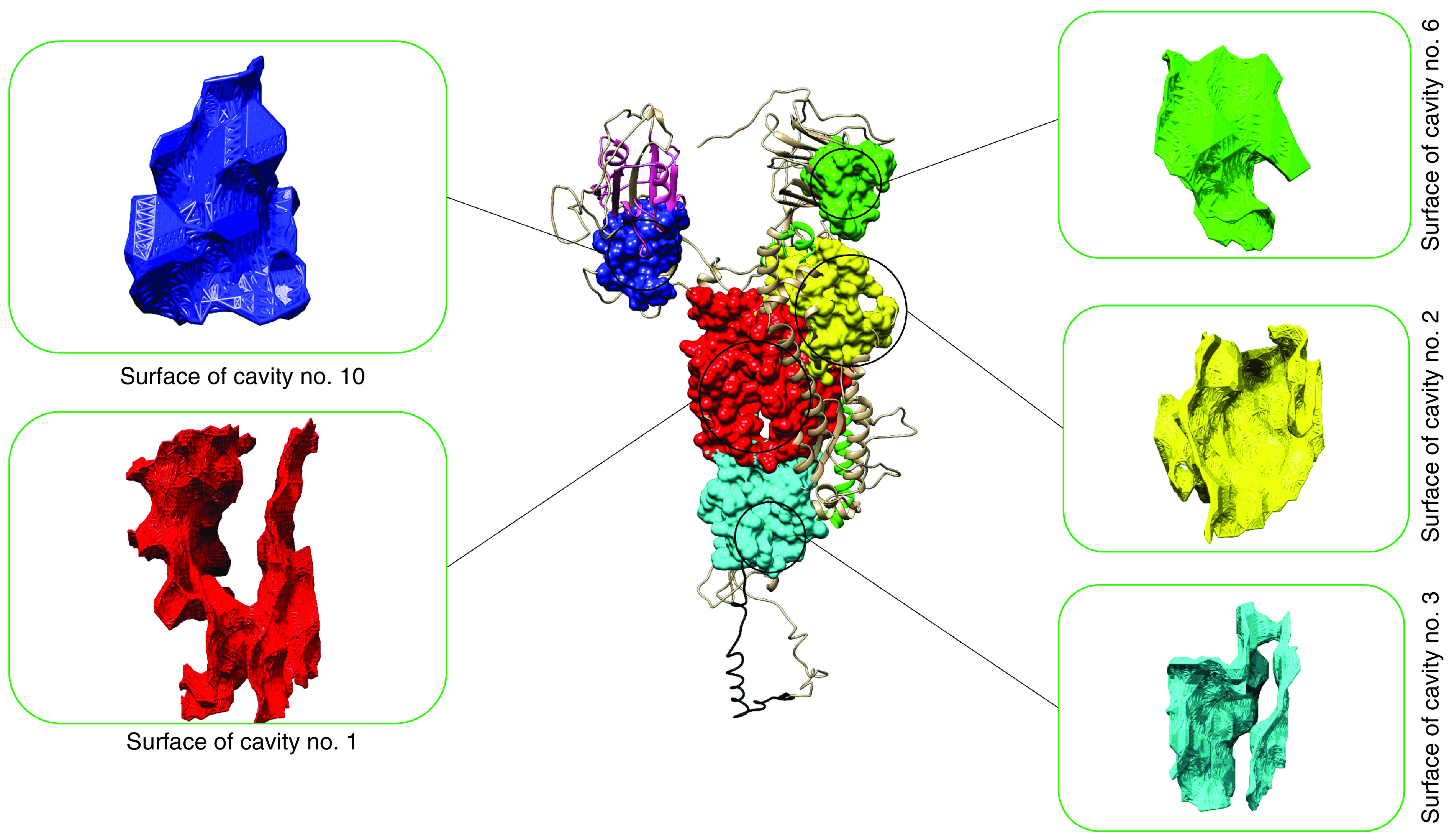
Illustration of five druggable cavity pockets within the monomeric SARS-CoV-2 spike glycoprotein. Pocket No.10 (shown in red) was the largest cavity and selected for pharmacophore modeling. Receptor-binding domain (residues 331–438) is represented in pink. In addition, heptad repeats 1 (HR1) and 2 (HR2) are depicted in light green and black, respectively.

A total of 42 cavities were found within the virus spike glycoprotein and five of them were druggable (Figure 1). The druggable cavity No.10 with highest drug score (10041) was selected for pharmacophore modeling. The residues within the cavity No.10 are provided in Supplementary Table 2.

### Pharmacophore of druggable cavity No. 10

**Figure 2.**
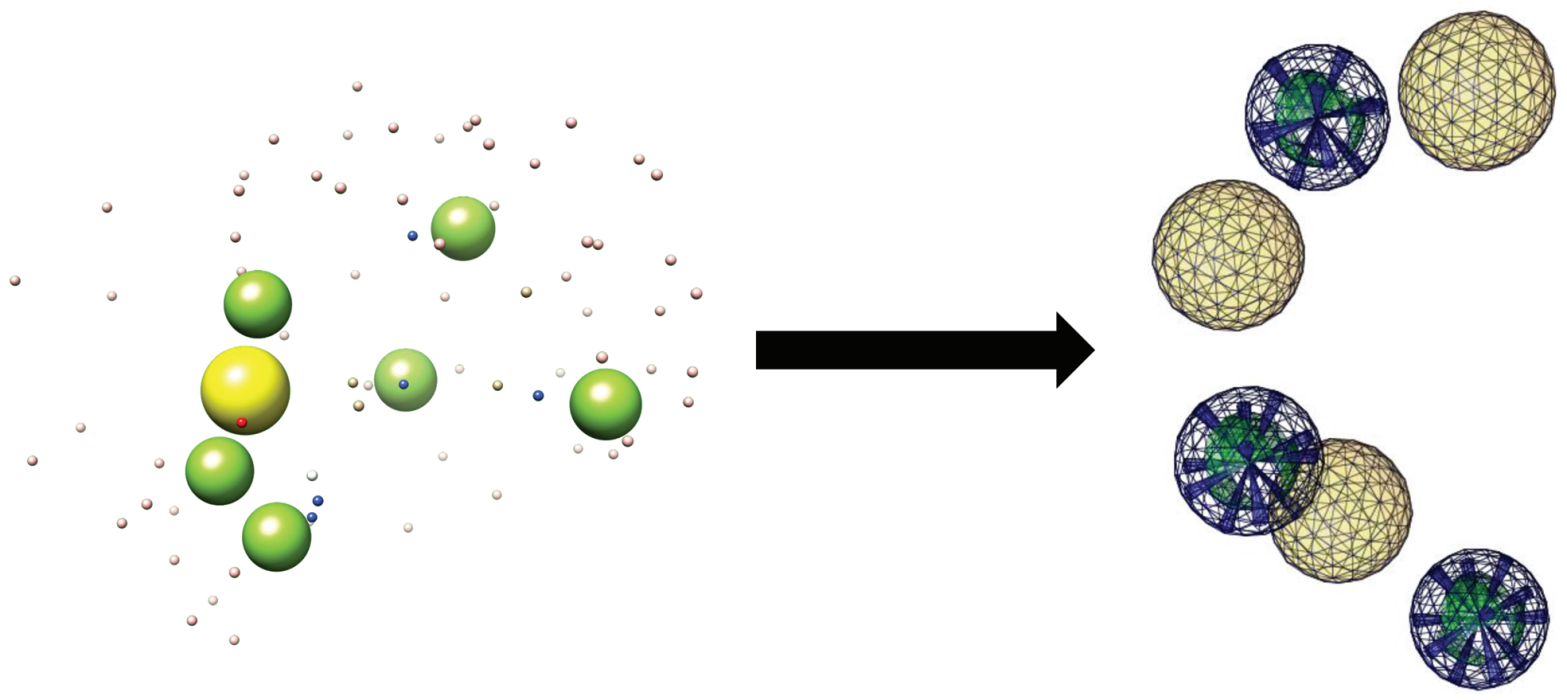
The resulted pharmacophore features and the extracted pharmacophore. **(Left)** The primary pharmacophore comprised of one positive ion center, one negative ion center, seven hydrogen donor sites, seven hydrogen acceptor sites and six hydrophobic regions and **(Right)** shows final pharmacophore made of three hydrophobic regions along with three positive ion regions.

**Figure 3.**
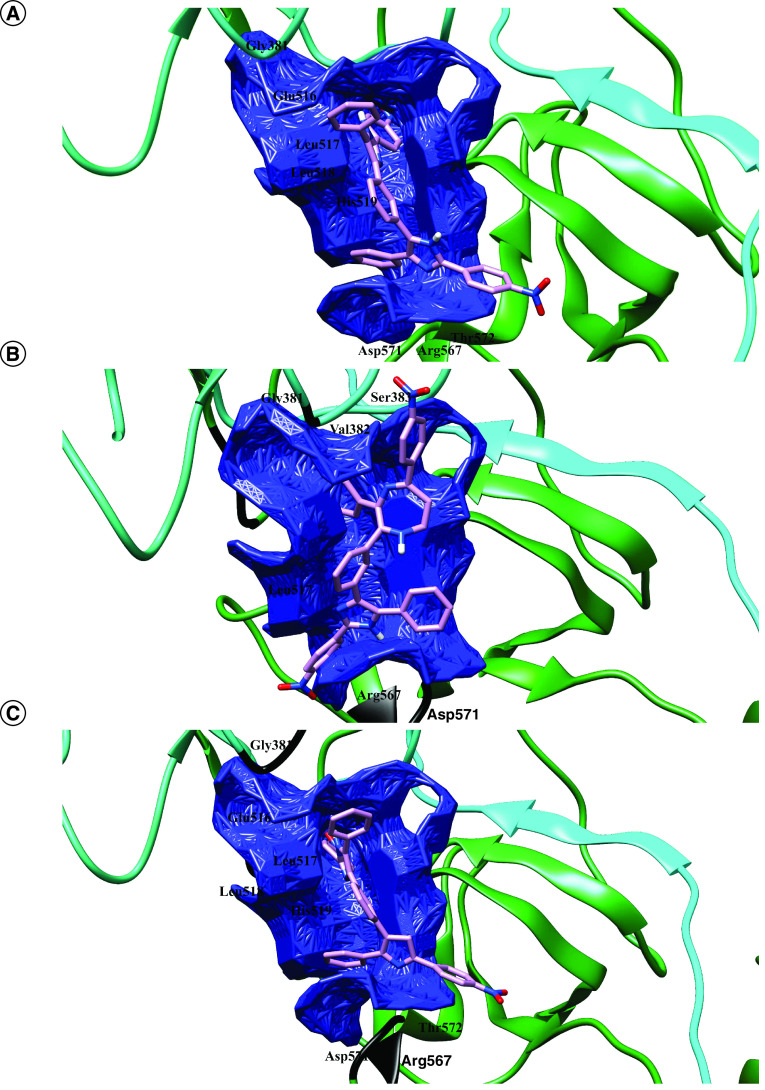
The druggable cavity pocket No. 10 and the lead compounds resided within the cavity. The figure illustrates binding sites of **(A)** Compound 38, **(B)** derivatives N4 to CCN and **(C)** N4 to H within cavity No. 10 in the SARS-CoV-2 S protein. The adjacent residues are also demonstrated.

As it is shown in the Figure 2, a pharmacophore was modeled with several features. For increasing the chance of hit identification, features of pharmacophore were reduced in a stepwise manner until at least 100 hits were identified. Of more than 1 billion compounds from ZINCPharmer and Pharmit, 410 hits were obtained to match with the created pharmacophore (Supplementary material, Hits.sdf). Following the Autodock Vina screening of the hits within the SARS-CoV-2 Spike glycoprotein, one lead (compound 38) with the highest affinity to the virus protein was discovered (-10.0 Kcal.mol^1^). The amino acids involved in interaction with the lead compound were G381, E516, L517, L518, H519, R567, D571 and T572. Of these residues, E516, L517 and D571 were at close contact (Figure 3).

### Screening of hit compounds & molecular docking

**Figure 4.**
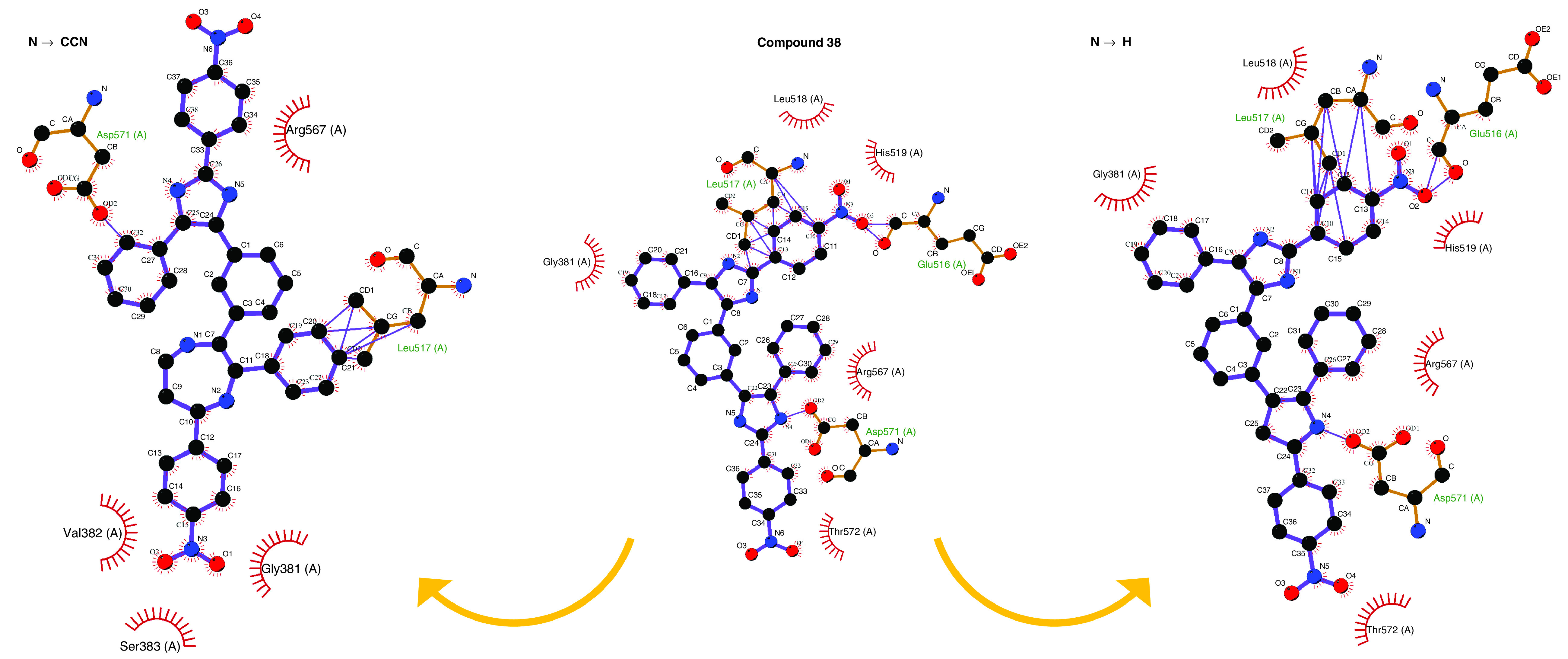
The rotation of the compound 38 derivatives within SARS-CoV-2 spike glycoprotein. Residues Leu517 and Asp571 are crucial at the interaction site of the compounds.

According to the identified residues within the viral glycoprotein, Compound 38 was used as a backbone for building chemical compound libraries (Table 1). In this regard, nitrogen number four (N4) in Compound 38 SMILES was substituted with ten functional groups (R^1^) to investigate the impact of N4 on Compound 38 affinity to S protein (Figure 4). As a result, N4 substitutions with CCN and H functional groups increased the affinity (-10.5 and -10.1 Kcal.mol^-1^, respectively) of the compound to the receptor. Drug-likeness and ADME properties of the ten molecules was predicted by SwissADME [43] and is provided in the Supplementary materials (Supplementary Table 1). Accordingly, no significant toxicity was predicted.

**Table 1.** Functional groups substituted with N4 of compound 38 and changes in the compounds' affinity to SARS-CoV-2 Spike glycoprotein.

Compounds (formula)	R^1^ substitutions	Affinity (Kcal.mol^-1^)
Compound 38 (C36H24N6O4)	c1Ncccc1	-8.9
	c1cNccc1	-9.4
	c1ccNcc1	-9.1
	Nc1ccccc1	-9.2
	Br	-9.1
	Cl	-9.4
	C	-10.0
	CCO	-10.0
	CCN	-10.5
	H	-10.1

## Discussion

Pandemic SARS-CoV-2 infection requires quick diagnosis and vaccine interventions. Potential drug targets for SARS-CoV-2 include spike glycoprotein, envelope protein, membrane protein, nucleocapsid protein (N), proteases, Nsp1, Nsp3 (Nsp3b, Nsp3c, Nsp3e), Nsp7 Nsp8 complex, Nsp9–Nsp10 and Nsp14–Nsp16, ORF7a, helicase and RdRp [13,14]. Several studies already worked on drug discovery of viral targets like 3CLpro, PLpro and RdRp [44,45], Mpro [45–53], Nsp3 [54], EndoU [55,56] and spike glycoprotein [57–59].

The spike glycoprotein plays an important role in virus entry [60]. S1 domain is as a major antigen on the surface of the virus that causes the initial interaction between the SARS-CoV-2 spike RBD and ACE2 receptor [17]. In addition, S2 domain causes viral–cell membranes fusion and so the virus enters the host body. Therefore, this protein can be considered as a potent drug target [20–22]. In the present study, SARS-CoV-2 spike glycoprotein was screened for druggable cavities. It was found that one major druggable cavity adjacent to the RBD domain of S glycoprotein can be used for pharmacophore-based drug discovery. Virtual library of natural products was used for the virtual screening.

Most of the investigated molecules are FDA-approved chemical drugs, hoping to highlight high affinity molecules in the context of drug repurposing [61]. Although some reports are promising, the chance of discovering novel drug candidates is not high due to the low number of chemicals within the drug-bank library [62]. Therefore, we used virtual screening of a library containing a large number of natural products to improve the odds of finding target-specific hits. Molecular docking was further used for screening and finding compounds with higher affinities for the SARS-CoV-2 spike protein. One chemical compound (compound 38) was identified as a potent inhibitor for blocking SARS-CoV-2. The results of this *in silico* study suggest that compound 38 might be able to interfere with RBD attachment to human ACE2 receptor. Data were further validated by molecular docking of compound 38 to energy minimized crystallographic structure of S glycoprotein (PDB ID: 6VXX) [63]. The affinity of compound 38 to the S protein was -9.3 Kcal.mol^-1^ and its binding site was the same as predicted in this study (data are not shown).

Two derivative compounds (H and CCN) showed promising interaction energies when functional group N4 of compound 38 was substituted with the functional groups. This indicates minor impact of this residue on compound 38 affinity to SARS-coV-2 S glycoprotein. *In vitro* research is being undertaken to determine the effectiveness of compound 38 on the viral propagation.

## Conclusion

This study presents a comprehensive search for significant druggable cavities within the SARS-CoV-2. In addition, millions of natural products have been screened. A chemical candidate was highlighted to block viral entry by interacting with the binding domain of viral spike glycoprotein. The findings of the study presented may be used in future studies on COVID-19 therapy.

**Summary points**

- SARS-CoV-2 spike glycoprotein is a main target for blocking the virus attachment process.
- SARS-CoV-2 spike glycoprotein was searched for druggable cavities.
- The best druggable cavity was chosen for pharmacophore-based drug discovery of a library with more than 1 billion compounds.
- 410 hits were identified with good matching identity within druggable pharmacophore.
- Docking screenings showed one compound (Compound 38) with the highest affinity to the cavity.
