# Supplementary material for: Druggability of cavity pockets within SARS-CoV-2 spike glycoprotein and pharmacophore-based drug discovery
Source: Future Virol. 2021 Jun 1:10.2217/fvl-2020-0394. doi: 10.2217/fvl-2020-0394 (PMC8176656; doi:10.2217/fvl-2020-0394)
Supplement: Supplementary file 1 [file GraphicalAbs.pdf]

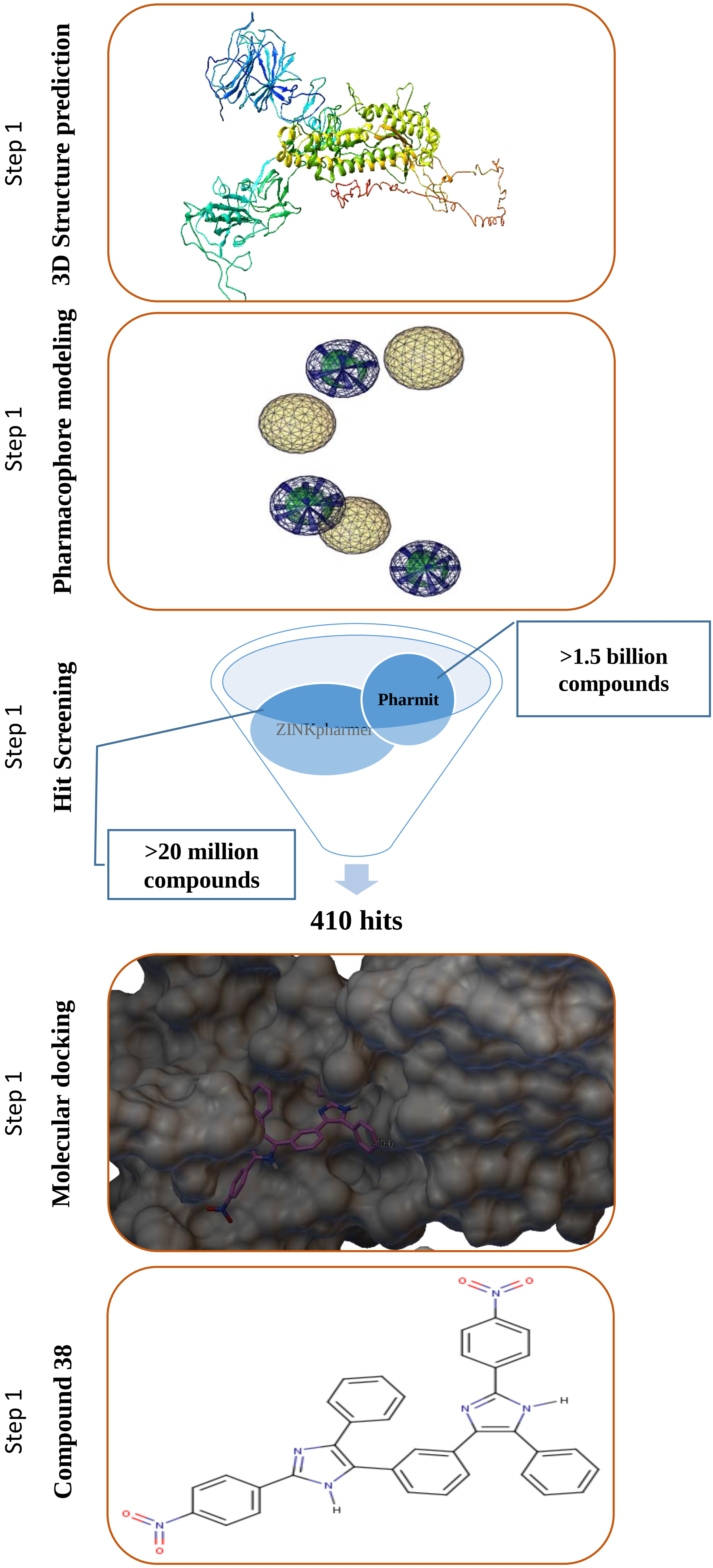
Step 1
3D Structure prediction
Step 1
Pharmacophore modeling
Step 1
Hit Screening
Pharmit
>1.5 billion compounds
ZINKpharmer
>20 million compounds
410 hits
Step 1
Molecular docking
Step 1
Compound 38
