# Supplementary material for: Druggability of cavity pockets within SARS-CoV-2 spike glycoprotein and pharmacophore-based drug discovery
Source: Future Virol. 2021 Jun 1:10.2217/fvl-2020-0394. doi: 10.2217/fvl-2020-0394 (PMC8176656; doi:10.2217/fvl-2020-0394)
Supplement: Supplementary file 3 [file Library.pdf]

Library. SMILES of 10 derivatives of Compound 38.

1. c1(cc(ccc1)C1=C(N=C(c1Ncccc11)c1ccc(cc1)[N+](=O)[O-])c1ccccc1)c1nc([nH]c1c1ccccc1)c1ccc(cc1)[N+](=O)[O-]
2. c1(cc(ccc1)C1=C(N=C(c1cNccc11)c1ccc(cc1)[N+](=O)[O-])c1ccccc1)c1nc([nH]c1c1ccccc1)c1ccc(cc1)[N+](=O)[O-]
3. c1(cc(ccc1)C1=C(N=C(c1ccNcc11)c1ccc(cc1)[N+](=O)[O-])c1ccccc1)c1nc([nH]c1c1ccccc1)c1ccc(cc1)[N+](=O)[O-]
4. c1(cc(ccc1)C1=C(N=C(Nc1ccccc11)c1ccc(cc1)[N+](=O)[O-])c1ccccc1)c1nc([nH]c1c1ccccc1)c1ccc(cc1)[N+](=O)[O-]
5. c1(cc(ccc1)C1=C(N=C(Br1)c1ccc(cc1)[N+](=O)[O-])c1ccccc1)c1nc([nH]c1c1ccccc1)c1ccc(cc1)[N+](=O)[O-]
6. c1(cc(ccc1)C1=C(N=C(Cl1)c1ccc(cc1)[N+](=O)[O-])c1ccccc1)c1nc([nH]c1c1ccccc1)c1ccc(cc1)[N+](=O)[O-]
7. c1(cc(ccc1)C1=C(N=C(C1)c1ccc(cc1)[N+](=O)[O-])c1ccccc1)c1nc([nH]c1c1ccccc1)c1ccc(cc1)[N+](=O)[O-]
8. c1(cc(ccc1)C1=C(N=C(CCO1)c1ccc(cc1)[N+](=O)[O-])c1ccccc1)c1nc([nH]c1c1ccccc1)c1ccc(cc1)[N+](=O)[O-]
9. c1(cc(ccc1)C1=C(N=C(CCN1)c1ccc(cc1)[N+](=O)[O-])c1ccccc1)c1nc([nH]c1c1ccccc1)c1ccc(cc1)[N+](=O)[O-]
10. c1(cc(ccc1)C1=C(N=C(H1)c1ccc(cc1)[N+](=O)[O-])c1ccccc1)c1nc([nH]c1c1ccccc1)c1ccc(cc1)[N+](=O)[O-]
