# Supplementary material for: Druggability of cavity pockets within SARS-CoV-2 spike glycoprotein and pharmacophore-based drug discovery
Source: Future Virol. 2021 Jun 1:10.2217/fvl-2020-0394. doi: 10.2217/fvl-2020-0394 (PMC8176656; doi:10.2217/fvl-2020-0394)
Supplement: Supplementary file 5 [file Table_S2.docx]

**Table S2.** The neighboring amino acid residues of the cavity number 10.

| **Cavity No.** | **Residues** |
| --- | --- |
| 10 | SER:46, VAL:47, LEU:48, HIS:49, THR:299, LYS:300, CYS:301, THR:302, LEU:303, LYS:304, SER:305, PHE:306, THR:307, VAL:308, GLU:309, LYS:310, GLY:311, ILE:312, TYR:313, GLN:314, THR:315, SER:316, ASN:317, PHE:318, SER:591, PHE:592, GLY:593, GLY:594, VAL:595, SER:596, THR:599, PRO:600, GLY:601, THR:602, ASN:603, THR:604, TYR:612, GLN:613, ASP:614, VAL:615, ASN:616, THR:645, ARG:646, ALA:647, GLY:648, CYS:649, TYR:660, GLU:661, CYS:662, ASP:663, ILE:664, PRO:665, ILE:666, GLY:667, ALA:668, GLY:669, ILE:670, CYS:671, ALA:672, SER:673, TYR:674, ARG:682, THR:696, MET:697, SER:698, LEU:699, GLY:700, ALA:701, GLU:702, VAL:722, THR:723, THR:724, GLU:725, ILE:726, LEU:727, PRO:728, MET:731, THR:732, ASN:824, LYS:825, VAL:826, THR:827, LEU:828, ALA:829, PHE:833, ASN:856, LEU:858, VAL:860, LYS:933, ILE:934, ASP:936, SER:937, LEU:938, SER:940, THR:941, ALA:942, SER:943, ALA:944, LEU:945, GLY:946, LYS:947, LEU:948, GLN:949, ASP:950, VAL:951, VAL:952, ASN:953, GLN:954, ASN:955, ALA:956, GLN:957, ALA:958, LEU:959, ASN:960, THR:961, LEU:962, VAL:963, LYS:964, GLN:965, GLN:1002, SER:1003, LEU:1004, GLN:1005, THR:1006, TYR:1007, VAL:1008, THR:1009, GLN:1010, GLN:1011, LEU:1012, ILE:1013, ARG:1014, ALA:1015, ALA:1016, GLU:1017, ILE:1018, ARG:1019, ALA:1020, SER:1021, ALA:1022, ASN:1023, LEU:1024, ALA:1025, THR:1027, LYS:1028, ARG:1039, VAL:1040, ASP:1041, PHE:1042, CYS:1043, GLY:1044, LYS:1045, PHE:1062, HIS:1064, ILE:1225, ALA:1226, ILE:1227, VAL:1228, MET:1229, VAL:1230, THR:1231, MET:1233, LEU:1234, CYS:1235, CYS:1236, MET:1237, THR:1238, SER:1239, CYS:1240, CYS:1241, SER:1242, LEU:1244, LYS:1245, GLY:1246, CYS:1247, CYS:1248, CYS:1250, GLY:1251, CYS:1253, CYS:1254, LYS:1255, PHE:1256, ASP:1257, GLU:1258, ASP:1260, SER:1261, GLU:1262, PRO:1263, VAL:1264, LEU:1265, LYS:1266, GLY:1267, VAL:1268, LYS:1269, LEU:1270, HIS:1271, TYR:1272, THR:1273 |
